# A functional dissociation of the left frontal regions that contribute to single word production tasks

**DOI:** 10.1016/j.neuroimage.2021.118734

**Published:** 2021-12-15

**Authors:** Justyna O. Ekert, Diego L. Lorca-Puls, Andrea Gajardo-Vidal, Jennifer T. Crinion, Thomas M.H. Hope, David W. Green, Cathy J. Price

**Affiliations:** a Wellcome Centre for Human Neuroimaging, UCL Queen Square Institute of Neurology, 12 Queen Square, London WC1N 3AR, United Kingdom; b Department of Speech, Language and Hearing Sciences, Faculty of Medicine, Universidad de Concepcion, Concepcion, Chile; c Faculty of Health Sciences, Universidad del Desarrollo, Concepcion, Chile; d Institute of Cognitive Neuroscience, University College London, London, United Kingdom; e Department of Experimental Psychology, University College London, London, United Kingdom

## Abstract

Controversy surrounds the interpretation of higher activation for pseudoword compared to word reading in the left precentral gyrus and pars opercularis. Specifically, does activation in these regions reflect: (1) the demands on sublexical assembly of articulatory codes, or (2) retrieval effort because the combinations of articulatory codes are unfamiliar? Using fMRI, in 84 neurologically intact participants, we addressed this issue by comparing reading and repetition of words (W) and pseudowords (P) to naming objects (O) from pictures or sounds. As objects do not provide sublexical articulatory cues, we hypothesis that retrieval effort will be greater for object naming than word repetition/reading (which benefits from both lexical and sublexical cues); while the demands on sublexical assembly will be higher for pseudoword production than object naming.

We found that activation was: (i) highest for pseudoword reading [P>O&W in the visual modality] in the anterior part of the ventral precentral gyrus bordering the precentral sulcus (vPCg/vPCs), consistent with the sublexical assembly of articulatory codes; but (ii) as high for object naming as pseudoword production [P&O>W] in dorsal precentral gyrus (dPCg) and the left inferior frontal junction (IFJ), consistent with retrieval demands and cognitive control.

In addition, we dissociate the response properties of vPCg/vPCs, dPCg and IFJ from other left frontal lobe regions that are activated during single word speech production. Specifically, in both auditory and visual modalities: a central part of vPCg (head and face area) was more activated for verbal than nonverbal stimuli [P&W>O]; and the pars orbitalis and inferior frontal sulcus were most activated during object naming [O>W&P]. Our findings help to resolve a previous discrepancy in the literature, dissociate three functionally distinct parts of the precentral gyrus, and refine our knowledge of the functional anatomy of speech production in the left frontal lobe.

## 1 Introduction

The left frontal lobe plays a well-researched role in speech production (Basilakos et al., 2018; Flinker et al., 2015; Long et al., 2016; Mugler et al., 2018). However, there is controversy as to the specific roles that distinct left frontal regions play in the generation of a speech plan. For example, as detailed below, some studies have associated the assembly of sublexical articulatory codes (e.g. phonemes and syllables) with activation in the left dorsal precentral gyrus, whereas others have claimed that sublexical assembly is supported by a more ventral region of the precentral gyrus (see Table 1). Here we consider the challenges of assigning specific functions to discrete regions and tackle this problem by using a multi-factorial design that enables us to tease apart the demands on articulatory planning from more general, non-linguistic processes such as working memory, attention and cognitive control.

**Table 1:** Literature review.

Activation	Baseline	First Author (date)	MNI Coordinates	pOp	PCg
**Reading aloud**					
Pseudowords	Regular words (consistent spelling-sound mappings)	Fiez et al. (1999)	−51, 14, 8*^	67%	5%
		Herbster et al. (1997)	−44, 4, 16	11%	10%
		Mechelli et al. (2005)	−54, 8, 18	38%	44%
		Carreiras et al. (2007)	−46, 8, 28	33%	33%
		Binder et al. (2005)	−43, 2, 27	11%	45%
		Carreiras et al. (2007)	−56, 0, 34	–	69%
		Mei et al. (2014)	−52, 0, 40	–	69%
	Words	Mechelli et al. (2003)	−48, 8, 22	35%	25%
		Brunswick et al. (1999)	−48, 6, 26	38%	36%
	Irregular words (inconsistent spelling-sound mappings)	Binder et al. (2005)	−51, 2, 13	5%	34%
		Binder et al. (2005)	−48, 0, 28	–	42%
		Mechelli et al. (2005)	−56, 0, 40	–	83%
Irregular words (inconsistent spelling-sound mappings)	Regular words (consistent spelling-sound mappings)	Herbster et al. (1997)	−46, 6, 16	23%	17%
		Mechelli et al. (2005)	−52, 2, 18	–	46%
		Binder et al. (2005)	−50, 7, 21	27%	41%
		Binder et al. (2005)	−51, 0, 36	–	65%
		Binder et al. (2005)	−44, −4, 43	–	37%
**Word matching**					
Syllables	Semantic	Poldrack et al. (1999)	−47, 0, 13	–	–
		Price et al. (1997)	−52, −2, 24	–	40%
		Devlin et al. (2003)	−50, 6, 24	28%	43%
		Devlin et al. (2003)	−42, 0, 28	–	50%
		Yen et al. (2019)	−52, 4, 30	6%	54%
		Mummery et al. (1998)	−52, −8, 38	–	37%
Rhyme	Semantic	Yen et al. (2019)	−50, 3, 30	5%	64%
	Synonym	Roskies et al. (2001)	−49, 3, 16	17%	31%
			−49, 1, 26	–	43%
**Attention to:**					
Phonology	Semantics	McDermott et al. (2003)	−55, 3, 15	–	65%
**Lexical decision**					
Pseudowords	Words	Fiebach et al. (2002)*	−49, 12, 12	45%	–
Words Sequential	Words Simultaneous	Twomey et al. (2015)	−57, 17, 7	62%	5%
			−51, 8, 22	28%	42%
			−54, 4, 43	–	48%
**Perception decision**					
Words (after assembled training)	Words (after addressed training)	Mei et al. (2014)	−56, 6, 24	13%	55%
Pseudowords	Words		−48, 6, 18	29%	36%

Left precentral gyrus (PCg) and pars opercularis (pOp) activation associated with sublexical processing in past studies - grouped by: task, activation condition, baseline condition and MNI z co-ordinate (ventral to dorsal). The Harvard-Oxford atlas (Desikan et al., 2006) was used to indicate the likelihood that the peak co-ordinates were in pOp or PCg. *Coordinates mapped from Talairach to MNI space using BioImage Suite (Lacadie et al., 2008).^ This effect was not observed in Fiez et al. (1999) when pseudowords were compared to low frequency consistent words (or low or high frequency inconsistent words).

From an extensive literature review (see Table 1 for details), we note that the majority of the functional neuroimaging studies investigating neural processing related to sublexical assembly compared activation for reading unfamiliar “pseudowords” to reading familiar words. Pseudowords (e.g. pholat) can only be read successfully by applying sublexical spelling to sound associations (e.g. ph+o + l + a + t or ph+ol+at or pho+lat). In contrast, reading familiar words (e.g. photos) is not dependant on sublexical assembly because it is facilitated by lexical (i.e. whole-word) knowledge. Thus, although reading words and pseudowords both involve the conversion of orthographic input into articulatory codes, the demands on integrating sublexical articulatory codes are higher when reading pseudowords.

A critical limitation of this approach is that enhanced activation for reading pseudowords compared to familiar words may not necessarily reflect greater demands on sublexical assembly. Instead, activation may reflect slower, more demanding speech production when the stimulus is unfamiliar. Indeed, the results detailed in Table 1 illustrate the similarity between the peak co-ordinates reported for reading aloud (A) pseudowords compared to words and (B) familiar words with “irregular” spelling-to-sound correspondences that are “inconsistent” with other words in the same language (e.g. yacht which is pronounced “yot” not “yatched”) compared to “regular” spelling-to-sound correspondences that are “consistent” with most other words in the same language (e.g. mint, hint, tint, flint, stint, print, splint). A plausible explanation is that this common activation reflects the demands on executive control (Fiez et al., 1999) because, in both cases, there is a conflict between lexical and sublexical processing – and the reader therefore has to attend to one and inhibit the other. For example, when reading the word “yacht”, the sublexical spelling-to-sound association (“yatched”) is inconsistent with the lexical spelling-to-sound association (“yot”). The output from sublexical assembly (“yatched”) therefore needs to be inhibited. Conversely, when reading the pseudoword “chiden”, the reader must inhibit the production of real words that look alike (e.g. children and chicken). For regularly spelled words, the demands on executive control are less because lexical and sublexical codes are, by definition, consistent.

Several studies have attempted to dissociate processing related to sublexical assembly and generic processing demands during speech production, but the conclusions have been inconsistent. For example, Fiez et al. (1999) and Mechelli et al. (2005) found that, compared to regular words, reading pseudowords and irregularly spelled words increased activation in the vicinity of the pars opercularis (Table 1), consistent with generic demands on mapping orthography-to-phonology, as opposed to sublexical assembly. In contrast, Mei et al. (2014) and Twomey et al. (2015) showed that activation at the same site (in standard space) is involved in sublexical assembly even when response times (reflective of general processing demands) are controlled. The role of the left dorsal precentral gyrus is also unclear. While Mechelli et al. (2005) and Twomey et al. (2015) associated it with sublexical processing; Binder et al. (2005) reported increased activation in this region for irregular than regular word reading, which is more consistent with generic demands. Further investigation is therefore required to understand these inconsistent conclusions.

In the current study, we considered how areas that were more activated for pseudoword than word production responded during object naming. Considering their response to object naming provides three advantages. First, object naming relies on lexical retrieval of articulatory codes and can be compared to reading and repeating the same object names, thereby controlling for speech output. Second, it is slower and more attention demanding than word reading (Glaser and Glaser, 1989), allowing us to segregate activation related to: (i) generic processing demands (object naming and pseudoword reading > word reading), (ii) sublexical assembly (pseudoword reading > object naming); (iii) lexical retrieval (object naming > pseudoword reading); and (iv) phonological-to-articulatory recoding (words and pseudowords > object naming). Third, the perceptual parts of pictures or sounds of objects do not provide any sublexical cues as to how the name is pronounced. This contrasts to irregular word reading, where high activation may reflect automatic but unsuccessful attempts at sublexical assembly. Finally, by including the corresponding conditions in the auditory modality (repetition of heard words and pseudowords, and naming objects from their sounds), we can dissociate activation related to articulatory planning from activation related to modality-specific processing (e.g. that related to mapping orthography onto phonology).

In summary, our literature review (Table 1) highlights a lack of clarity in how activation in and around the dorsal versus ventral left precentral gyrus contributes to speech production. Using a multi-factorial fMRI design, we investigated which parts of the left precentral gyrus were most consistent with: (1) the demands on sublexical assembly of articulatory codes (assumed to be higher for pseudoword reading than object naming) or (2) retrieval effort (assumed to be higher for object naming and pseudoword production than word production). Although our questions concern regions in the left frontal lobe, we also examined whole brain activation to delineate the neural networks in which different left frontal regions participate.

## 2 Methods

The data used in this paper have previously been reported in Oberhuber et al. (2016) where the goal was to dissociate the function of different parts of the left supramarginal gyrus. Here we focused on teasing apart how distinct left frontal lobe regions contribute to speech production.

### 2.1 Experimental design

**Fig. 1:**
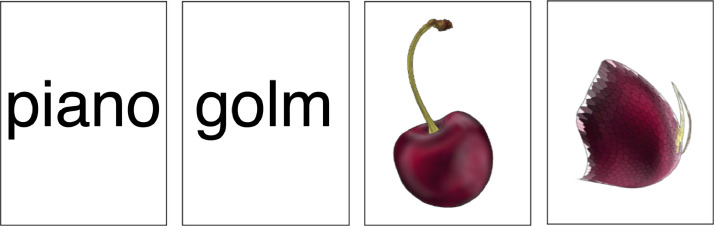
*Examples of visual stimuli*. Verbal (words/pseudowords) and nonverbal (pictures of objects and non-objects) visual stimuli.

There were 8 conditions that comprised a 2 × 2 × 2 factorial design (Table 2). Factor I was stimulus modality (auditory versus visual); Factor II was verbal versus nonverbal stimuli (words and pseudowords versus objects and baseline stimuli); Factor III was the presence or absence of semantic content (familiar words and object names versus unfamiliar pseudowords and baseline stimuli). Examples of the visual stimuli are shown in Fig. 1. Each condition was presented in a separate run, with blocks of stimuli alternating with rest. Full details of the experiment (e.g. regarding stimulus selection) can be found in Oberhuber et al. (2016).

**Table 2:** Experimental design.

Factor I	Stimulus		Factor II	Factor III
Input			Verbal vs.	Semantic vs.
			Nonverbal	Nonsemantic
Visual	Written object names	W	✓	✓
	Written pseudowords	P	✓	✗
	Pictures of objects	O	✗	✓
	Coloured patterns	B	✗	✗
Auditory	Heard object names	W	✓	✓
	Heard pseudowords	P	✓	✗
	Sounds of objects	O	✗	✓
	Humming (male or female voice)	B	✗	✗

**Factor IV** = Task: Speech production or 1-back matching.

Key: W= words, P = pseudowords, O = objects, B = baselines.

### 2.2 Participant groups

There were two non-overlapping participant groups (*n* = 25 and 59) that both performed the same 8 tasks of interest embedded within one of two different experimental paradigms. In addition to the 8 speech production conditions examined in the current analysis, Group 1 completed 1-back matching tasks on the same 8 stimulus sets; while Group 2 completed 5 tasks that involved sentence production, verb production, noun production and semantic decisions on pictures of objects or their heard object names. These additional tasks were presented in separate scanning sessions and were not examined in the current analysis. Although the presentation parameters in the two paradigms were not exactly the same (see Table 3), our focus is on results that were observed across both datasets. Direct comparison of the same effects in Group 1 and Group 2, did not reveal any significant differences.

**Table 3:** Experimental details for: Group 1 Group 2.

	Group 1	Group 2
**Participants**		
Number	25	59
Gender (n females/ n males)	12/13	34/25
Mean age in years (+/−SD)	31.44 (5.74)	44.5 (17.66)
**Stimulus properties**		
**Stimulus duration in sec (+/−SD)**		
Visual stimuli	1.5	2.5
Auditory words	0.64 (0.10)	0.63 (0.09)
Auditory pseudowords	0.68 (0.12)	0.65 (0.08)
Sounds	1.47 (0.12)	1.45 (0.15)
Hums	1.04 (0.43)	1.05 (0.51)
**Average number of syllables (SD)**		
Reading words	1.53 (0.68)	1.55 (0.68)
Repeating words	1.53 (0.68)	1.68 (0.73)
Reading pseudowords	1.94 (0.92)	1.50 (0.51)
Repeating pseudowords	1.90 (0.84)	1.50 (0.51)
Naming pictures	1.55 (0.69)	1.48 (0.72)
Naming sounds	1.81 (0.92)	1.88 (0.94)
Naming gender	1.50 (0.51)	1.50 (0.51)
Naming colours	1.36 (0.49)	1.40 (0.50)
**Average number of letters (+/−SD)**		
Reading words	5.24 (1.68)	5.08 (1.61)
Repeating words	5.24 (1.68)	5.28 (1.38)
Reading pseudowords	5.28 (1.94)	4.40 (1.03)
Repeating pseudowords	5.35 (1.72)	4.35 (1.08)
Naming pictures	5.30 (1.75)	5.28 (1.75)
Naming sounds	5.64 (2.21)	5.65 (2.40)
Naming gender	5.00 (1.01)	5.00 (1.01)
Naming colours	4.89 (1.04)	4.80 (1.18)
**Timing parameters**		
ISI (sec)	2.52	2.5
Number of stimuli per block	9 (& 1 repeat)	10
Number of stimulus blocks per run	4	4
Total number of stimuli per run	36	40
Number of runs included/total	8/16	8/13
Total time for each run (min)	3.2	3.4
Total acquisition time (min)	25.6	27.2
**Scanning parameters**		
TR (sec)	3.085	3.085
Number of slices	44	44
Number of volumes per run	62	66

### 2.3 Counterbalancing

In Paradigm 1 (*n* = 25), the same object concepts were rotated across the 4 semantic conditions – either as written object names, heard object names, pictures of objects or sounds of objects. In addition, written pseudowords were matched to spoken pseudowords. This ensured that the speech being produced was the same for the matched conditions (across subjects). The order of conditions was counterbalanced across participants in Group 1. In Group 2 (*n* = 59), we used a fixed condition order so that inter-subject variability could not be attributed to differences in condition order. The figures illustrating our results demonstrate that our effects of interest were observed in both groups – which further strengthens our conclusions. Table 3 provides participant, experimental and scanner details for each group of subjects.

### 2.4 fMRI data preprocessing

Data preprocessing and statistical analysis were performed in SPM12 (Wellcome Centre for Human Neuroimaging, University College London, UK), running on MATLAB 2012a. Functional volumes were spatially realigned to the first EPI volume and unwarped to compensate for non-linear distortions caused by head movement or magnetic field inhomogeneity. The unwarping procedure was used in preference to including the realignment parameters as linear regressors in the first-level analysis because unwarping accounts for non-linear movement effects by modelling the interaction between movement and any inhomogeneity in the T2* signal. After realignment and unwarping, the realignment parameters were checked to ensure that participants moved less than one voxel (3mm^3^) within each scanning run.

The anatomical T1w image was co-registered to the mean EPI image generated during the realignment step and then spatially normalised to the MNI space using the unified normalisation-segmentation routine in SPM12. To spatially normalise all EPI scans to MNI space, the deformation field parameters that were obtained during the normalisation of the anatomical T1w image were applied. The original resolution of the different images was maintained during normalisation (voxel size 1  × 1  ×  1 mm^3^ for structural T1w and 3 ×  3  ×  3 mm^3^ for EPI images). After normalisation, functional images were spatially smoothed with a 6 mm full-width-half-maximum isotropic Gaussian Kernel to compensate for residual anatomical variability and to permit application of Gaussian random-field theory for statistical inference (Friston et al., 1995).

### 2.5 First level statistical analyses

Each preprocessed functional volume was entered into a subject specific fixed effect analysis using the general linear model. Stimulus onset times were modelled as single events. For Paradigm 1 (Group 1), we used 2 regressors per task, one modelling instructions, and the other modelling each stimulus. For Paradigm 2 (Group 2), the stimulus regressor was replaced with three different regressors for correct, incorrect, and delayed/no responses, resulting in a total of 4 regressors per task. This is because Paradigm 2 was designed for patients who were expected to make errors. Importantly, the current study (with neurotypical participants) did not find significant differences between effects of interest in Paradigm 1 (activation across trials of the same stimulus type) and Paradigm 2 (activation related to correct trials only). This is not unexpected given the very low number of incorrect/no response trials in both groups. Stimulus functions were convolved with a canonical haemodynamic response function and high pass filtered with a cut-off period of 128 s.

For each scanning session/run (that alternated one condition of interest with fixation), we generated a single contrast that compared activation in response to the stimuli and task of interest to resting with fixation. This resulted in 16 different contrasts (one per condition) for each participant. Each contrast for each individual was inspected to ensure that there were no visible artefacts (e.g. edge effects, activation in ventricles) that might have been caused by within-scan head movements.

### 2.6 Second level statistical analysis

The first level analysis for each participant yielded 8 separate contrasts (one per condition > fixation), i.e. words (W), pseudowords (P), objects (O) and baseline (B) in the visual and auditory modality (see Table 2). The second level analysis modelled 16 conditions; 8 for each group of participants. Contrasts were computed across group and the consistency across groups is demonstrated in the Figures illustrating the results.

The effects of interest were: (1) the main effect of verbal compared to nonverbal stimuli (W&P > O&B); and (2) the interaction of verbal/nonverbal and semantic/nonsemantic (i.e. P&O>W&B). Post hoc tests were then used to segregate three different effects driving the interaction: Contrast A [P>W&O] segregated activation that was higher for pseudoword reading/repetition compared to word reading/repetition and object naming (i.e. consistent with the demands on sublexical assembly). We also expected that activation related to sublexical assembly would be higher for words than objects (i.e. P>W>O). Contrast B [P&O>W] segregated activation that was higher for object naming and pseudoword reading/repetition compared to word reading/repetition (consistent with generic retrieval demands). Contrast C [O>W&P] segregated activation that was higher for object naming compared to word reading/repetition and pseudoword reading/repetition. We did not include the baselines in these contrasts as this is less conservative (baselines put lower processing demands on sublexical processing and executive control) and our goal was to distinguish processing for P&W&O.

Each of these contrasts was repeated three times: once across modality, once in the visual modality and once in the auditory modality. If an effect was observed in one modality only, we checked and reported the interaction of that effect with the main effect of stimulus modality (visual versus auditory).

We report all results when the main contrast (see Table 2 and above) was significant at *p* < 0.05 after family-wise error correction in height. To ensure that the activation fitted the effect of interest, we used the inclusive masking option in SPM (thresholded at *p* < 0.05 uncorrected),  see Table 4A for details. The type of processing that we expected to be probed for each effect is provided in Table 4B and rationalised in the Discussion.

**Table 4:** Statistical contrasts and interpretations.

A: Contrasts used to isolate effects of interest											
		Main Contrast	Inclusive (✓)masks								
			P>W	P>O	W>P	W>O	O>W	O>P	P>B	O>B	W>B
ME	Verbal > nonverbal	W&P>O&B		✓		✓			✓		✓
A	Sublexical assembly	P>W&O	✓	✓		✓			✓		✓
B	Retrieval demands	P&O>W	✓				✓		✓	✓	
C	Highest for naming	O>W&P					✓	✓		✓	


B: Interpretation											
Effect of interest		Main Contrast	Type of processing that might be probed								
ME	Verbal > nonverbal	W&P>O&B	Phonological-to-articulatory recoding								
A	Sublexical assembly	P>W&O	Sublexical assembly of articulatory plans (g-o-l-m)								
B	Retrieval demands	P&O>W	Highest demands on retrieving articulatory plans								
C	Highest for naming	O>W&P	Retrieving whole word articulatory plans								

Key: ME = main effect, W = words, P = pseudowords, O = objects, B = baseline.

✓ Inclusive masks (visual &/or auditory).

**Table 5:** Left frontal regions associated sublexical assembly, retrieval demands, and naming.

Effect of interest		Main contrast	x	y	z	Vx	Z-scores		Location
							Main	Int.	
									
A	Sublexical assembly	P>W&O	−57	9	18	30	5.7	4.9	Ventral precentral sulcus/gyrus
			−54	6	27		5.9	5.5	
			−51	0	33		4.8	4.9	
B	Retrieval demands	P&O>W	−39	6	27	88	>8	8.1	Inferior frontal junction
			−48	3	48		5.4	7.1	Dorsal precentral gyrus
C	Highest for naming	O>W&P	−39	15	27	90	>8	5.7	Inferior frontal sulcus
			−45	30	15		7.3	>8	
			−30	33	−9	75	7.6	7.3	Pars orbitalis
			−30	27	3		>8	>8	

W = words, P = pseudowords, O = objects, Int. = interaction of semantics and verbal input, Vx = number of contiguous voxels at *p* < 0.001 uncorrected. All effects were significant after voxel-level correction for multiple comparisons across the whole brain.

## 3 Results

### 3.1 Behavioural results

**Fig. 2:**
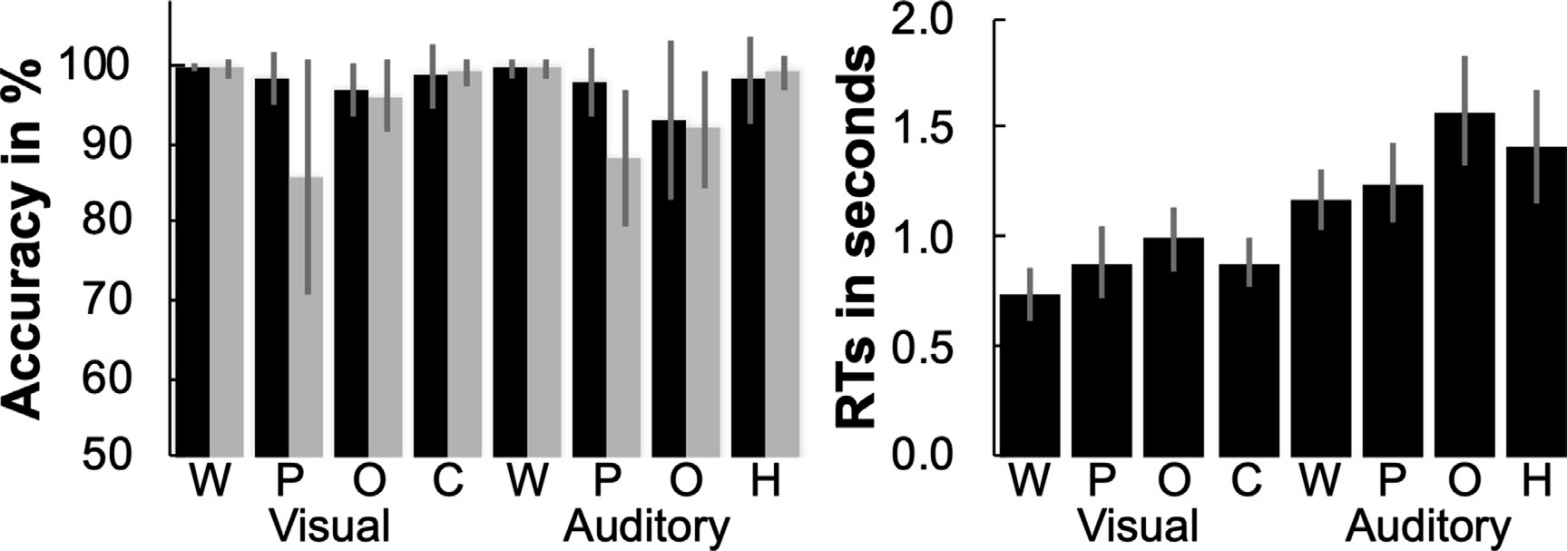
*In-scanner behavioural scores*. Task specific accuracy for Group 1 (grey plots) and Group 2 (black plots, n = 58 following removal of 1 outlier) and response times (RTs) for Group 2 only (*n* = 57 following exclusion of 2 subjects with missing RT data due to technical failure). Plots show mean scores with standard deviation (SD) as red bars. W = words, P = pseudowords, O = objects, C = colours (visual baseline), H = humming sounds (auditory baseline).

Details of the in-scanner behavioural performance for our participants are illustrated in Fig. 2 and reported in Oberhuber et al. (2016). Accuracy scores for Experiment 2 were computed after two outliers (subjects with less than 50% accuracy) had been removed. In brief, the average in-scanner accuracy was 95% for Group 1 and 98% for Group 2. Response times (RTs) were only available for Group 2 (due to technical failure in Group 1) and were computed after two participants were excluded due to missing RT data. Across modality, RTs were slower for auditory than visual speech production stimuli due to the sequential delivery of each auditory stimulus, in contrast to the simultaneous delivery of all parts of each visual stimulus. Within modality, participants were slower on more demanding tasks, specifically: (a) object naming than word repetition or reading, consistent with object naming being more demanding; (b) object naming than pseudoword production, and (c) pseudowords than words with this effect trading with less accurate pseudoword production than object naming.

### 3.2 fMRI results

**Fig. 3:**
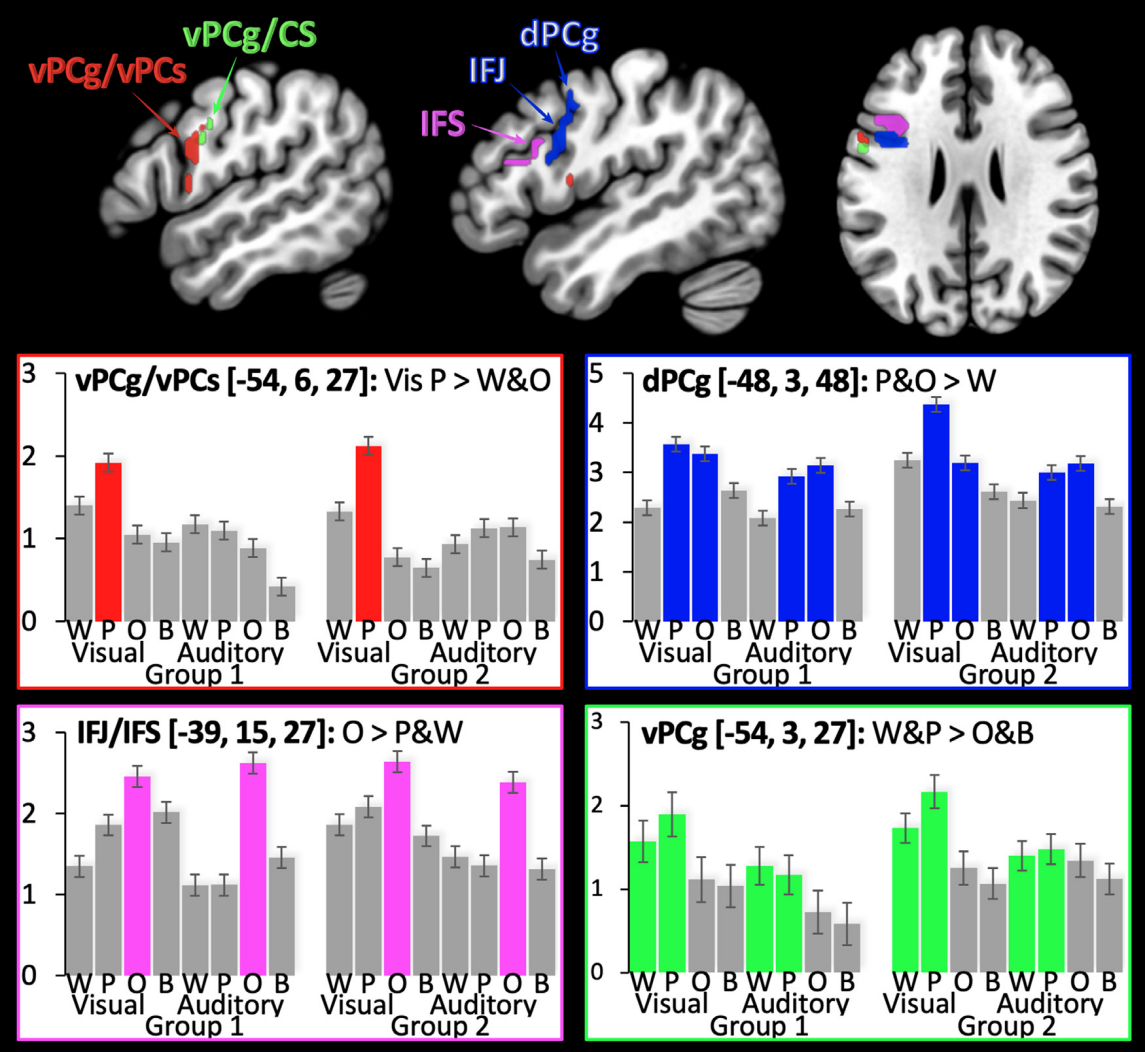
*Anatomical location of effects of interest and their condition dependant responses*. Relative location of each effect shown on a standard structural template in MNI space at slices x= −48, x= −54, z= +27. The estimated effect size is illustrated for Words (W), Pseudowords (P), Object naming (O) and Baseline conditions (B) in the visual (columns 1–4 and 9–12) and auditory modalities (columns 5–8 and 13–16). Columns 1–8 are from Group 1. Columns 9–16 are from Group 2. The coloured bars highlight the activation conditions. The error bars are standard error. Although each effect of interest was highly significant, these plots show that there is high selectivity without specificity (i.e. all regions were activated across conditions). dPCg/vPCg/vPCs = dorsal/ventral precentral gyrus/sulcus; IFJ/IFS = Inferior frontal junction/sulcus. Regions associated with sublexical assembly (P>W&O) are shown in red; naming (O>W&P) in magenta; generic retrieval demands (P&O>W) in blue; verbal > nonverbal (W&P>O&B) in green (For interpretation of the references to color in this figure legend, the reader is referred to the web version of this article.).

Left frontal activation (in front of the central sulcus) was highly significant for the main effect of verbal > nonverbal stimuli (W&P>O&B) across stimulus modality. Peak activation [−54, +3, 27; Z-score= 6.2] was located in the left ventral precentral gyrus (head and face area; see Fig. 3). The interaction (P&O>W&B) between verbal/nonverbal and semantic/nonsemantic also yielded highly significant frontal activation that we segregated, with post hoc tests, into three different effects (A, B and C), as described below.

#### 3.2.1 Sublexical assembly (P>W&O)

Activation that was highest for pseudowords (P>W&O) was observed for visual stimuli only, in the anterior part of the left ventral precentral gyrus that borders the ventral precentral sulcus (Table 4; red in Fig. 3) with no corresponding effect in the auditory modality. This resulted in a modality by condition (P>W&O) interaction that was significant at *p* < 0.001 uncorrected: Z-scores = 4.1 at [−57, +9, +18]; 4.1 at [−54, +6, +27]; 4.2 at [−48, 0, +33]. Activation in vPCg/vPCs was, however, not specific to reading because it was greater for repeating words (*p* < 0.05 corrected), repeating pseudowords (*p* < 0.05 corrected), auditory object naming (*p* < 0.05 corrected), and gender naming (*p* < 0.005 uncorrected) than rest (see Fig. 3).

The same pattern of effects was also observed in the left anterior putamen (as reported in Oberhuber et al., 2013) and the left postcentral sulcus.

#### 3.2.2 Generic demands on articulatory planning (P&O>W)

Activation was higher for pseudowords and objects than words deep in the inferior frontal junction, extending laterally through the precentral sulcus to the dorsal precentral gyrus (Table 4; blue in Fig. 3), with no significant difference between the visual or auditory modalities (*p* > 0.05 uncorrected). The same response pattern (P&O>W) was also observed in the bilateral anterior insula/frontal operculum and pre-SMA.

#### 3.2.3 Highest for naming (O>W&P)

Activation was higher for objects than pseudowords and words in the left inferior frontal sulcus and left pars orbitalis (Table 4; magenta in Fig. 3), with no significant difference between the visual or auditory modalities (*p* > 0.05 uncorrected). The same response pattern (O>W&P) was also observed in the left middle temporal sulcus, left fusiform, bilateral visual cortices and bilateral cerebellum.

#### 3.2.4 Other left frontal lobe activation

No activation was detected in the precentral gyrus, precentral sulcus or pars opercularis for: the main effects of semantic > nonsemantic; nonsemantic > semantic; nonverbal > verbal; or auditory > visual. However, the main effect of visual > auditory stimuli identified left precentral activation [peak at −42, 3, 30] that was highest for reading pseudowords (effect A) and least for repeating words or gender naming.

## 4 Discussion

Prior studies have reported that increased demands on sublexical assembly of articulatory codes (e.g. phonemes and syllables) increases activation in either dorsal (Mechelli et al., 2005) or ventral (Mei et al., 2014; Twomey et al., 2015) parts of the left precentral gyrus (Table 1). However, possible confounds in the experimental designs of previous studies make it difficult to determine the type of processing that engages each region. To further dissociate the functional contribution of distinct left frontal regions to speech production, we compared activation for word and pseudoword production to that observed during object naming, which exerts high demands on the retrieval of whole-word articulatory plans.

Our results indicate that the response in the left ventral precentral gyrus (head and face area), bordering the ventral precentral sulcus (vPCg/vPCs), is most consistent with sublexical assembly of articulatory codes, because activation was higher for pseudoword reading than object naming and word reading. In contrast, we found that the response in the left dorsal precentral gyrus (dPCg) extending into the left inferior frontal junction (IFJ) is most consistent with retrieval demands, because activation was higher for object naming and pseudoword reading/repetition than word reading/repetition. This functional dissociation between ventral and dorsal parts of the precentral gyrus is consistent with the heterogeneity evidenced by multimodal connectivity-based parcellation (Genon et al., 2018).

Our multi-task approach also allowed us to dissociate other functionally distinct regions in the left frontal lobe that are differentially engaged during single-word speech production. Below, we discuss how each of our findings confirm, extend and challenge the results of previous studies, and their relevance for refining our understanding of the functional anatomy of speech production. A summary of the findings, and interpretation related to prior literature can be found in Table 6.

**Table 6:** How different left frontal regions may contribute to speech production.

Region	Prior hypotheses	Effect	Most parsimonious explanation
**vPCg/vPCs**	(a) Sublexical assembly of articulatory plans (b) Retrieval effort (c) Conflict resolution	P>W&O	(a) Sublexical assembly of articulatory plans
**dPCg**	(a) Sublexical assembly of articulatory plans (b) Retrieval effort/ executive functions (c) Conflict resolution	P&O>W	(a) Retrieval effort / executive control
**vPCg**	(a) Sublexical assembly of articulatory plans (b) Retrieval effort/ executive functions	W&P>O&B	Neither hypothesis confirmed We propose: “phonological-to-articulatory recoding”
**IFJ**	Cognitive control/ attention working memory	P&O>W	Consistent with prior hypothesis
**IFS**	(a) Word retrieval (b) Integration of information prior to response selection	O>W&P	(b) Integration of information prior to response selection
**pOrb**	Semantic retrieval	O>W&P	Semantic-to-articulatory recoding

dPCg/vPCg/vPCs: Dorsal/ventral precentral gyrus/sulcus.

IFJ/IFS: Inferior frontal junction/sulcus. pOrb: Pars orbitalis.

### 4.1 Sublexical assembly (P>W&O in the visual modality)

Left frontal activation associated with sublexical processing was identified on the anterior surface of the left ventral precentral gyrus (vPCg), bordering the ventral precentral sulcus. The MNI co-ordinates of peak activation in this area ([−57, 9, 18] and [−54, 6, 27]) corresponds to those associated with sublexical assembly in Mei et al. (2014) and Twomey et al. (2015) using completely different experimental designs. In Mei et al. (2014), native English speakers were trained to read words presented in unfamiliar Korean Hangul characters by either recognising the words as a whole or by relying on the sublexical spelling to sound relationships. When reading the same words in the scanner, those using a sublexical assembly strategy increased activation at MNI co-ordinates [−56, 6, 24] compared to those who read the words lexically. In Twomey et al. (2015), a very similar area (MNI co-ordinates [−51, 8, 22]) was more activated when words emerged on the screen sequentially compared to when they emerged as a whole.

Other reading studies (Binder et al., 2005; Mechelli et al., 2005) did not associate the vPCg with sublexical assembly because activation increased for words with irregular compared to regular spellings (see Table 1) and irregular spellings cannot be read successfully using sublexical assembly. Our alternative interpretation of the enhanced vPCg/vPCs response during irregular reading is that skilled readers will automatically engage sublexical assembly when presented with familiar orthography. Moreover, unsuccessful sublexical processing may persist for irregular word reading until the correct pronunciation is retrieved via lexico-semantics.

The vPCg activation we associate with sublexical processing was on the anterior surface of vPCg, bordering the ventral precentral sulcus. Here, cortical activity has been related to the motor planning of vocal tract actions required to produce speech sounds (articulatory gestures) at discrete times (Mugler et al., 2018). In this context, enhanced activation for pseudoword reading compared to word reading and object naming can be explained by enhanced demands on encoding novel sequences of articulatory gestures.

Although vPCg/vPCs activation was not enhanced for pseudoword repetition compared to word repetition and auditory naming, it was not specific to reading. Specifically, we also found highly significant vPCg/vPCs activation (*p* < 0.05 corrected) for repeating words and for repeating pseudowords (Fig. 3), consistent with the demands on articulatory planning that is independent of stimulus modality. The increased demands that pseudoword word reading places on articulatory planning can be explained by the absence of facilitation from (i) an auditory short-term representation of the intended speech output (Strand et al., 2008) that is available during auditory repetition; and (ii) the lexical/semantic familiarity associated with word reading.

### 4.2 Generic demands on articulatory planning (P&O>W)

The area associated with generic retrieval demands was located deep in the left frontal lobe, with one peak falling in the left inferior frontal junction (located at the junction of the inferior precentral sulcus and inferior frontal sulcus) and a second peak in the left dorsal precentral gyrus (dPCg). The inferior frontal junction (IFJ) is part of a network associated with attention, cognitive control and working memory (Cole and Schneider, 2007, Roth et al., 2006)(Roth et al., 2006; Cole and Schneider, 2007; Muhle-Karbe et al., 2016; Tamber-Rosenau et al., 2018; Zhang et al., 2018(Zhang et al., 2018)) that also includes the dorsolateral prefrontal cortex, anterior insula, and pre-SMA (Sundermann and Pfleiderer, 2012) - all regions that were co-activated with the IFJ in the current study (blue areas in Fig. 3).

The dPCg has previously been associated with sublexical assembly because it was more activated for reading pseudowords compared to reading irregularly and regularly spelled words (Mechelli et al., 2005); and for reading text delivered sequentially rather than simultaneously (Twomey et al., 2015). Our finding that activation was higher for object naming than word reading is not consistent with this claim. Instead, our findings are more consistent with prior studies that demonstrated a role for the left dPCg in retrieving fine-grained motor plans and anticipating rhythms (Chen et al., 2008) during speech articulation and finger movements (Meister et al., 2009); particularly when people watch/listen to material for which they have been highly trained to generate very specific action responses, including dance movements (Calvo-Merino et al., 2005), piano music (Lahav et al., 2007) and violin music (Dick et al., 2011). According to this hypothesis, left dorsal precentral activation should be lower when retrieval demands are lower (i.e. for reading and repeating words), as observed in the current study.

### 4.3 Highest activation for object naming (O>W&P)

In contrast, retrieving articulatory plans from semantic stimuli (i.e. semantic-to-articulatory recoding) enhanced activation in (i) the left pars orbitalis (pOrb), a region already associated with controlled semantic retrieval (Sabb et al., 2007), and (ii) the left inferior frontal sulcus, a region  already associated with word retrieval (Arya et al., 2019; Price, 2012). The left inferior frontal sulcus has also been associated with the integration of bottom-up and top-down multi-sensory information (semantic, nonsemantic and nonverbal) prior to response selection (Adam and Noppeney, 2010; Gau and Noppeney, 2016; Noppeney et al., 2010).

### 4.4 The main effect of verbal > nonverbal stimuli (W&P>O&B)

In a central part of vPCg, we found that activation was higher for verbal stimuli (words and pseudowords) than nonverbal stimuli (object, colour and gender naming) in both auditory and visual modalities (green in Fig. 3). As activation in this part of vPCg was not higher for pseudowords than words, it is not consistent with the expected demands on sublexical assembly of articulatory plans. We therefore propose that enhanced activation in the central part of vPCg for verbal more than nonverbal stimuli reflects the association of articulatory codes with phonological representations of the stimuli (as opposed to the subsequent assembly of these codes). Although further studies are required to investigate this hypothesis, we speculate that phonological-to-articulatory recoding may be evoked faster and sustained longer when processing verbal stimuli, compared to nonverbal stimuli because (i) we are highly trained to link verbal stimuli to their speech sounds and articulatory codes and (ii) nonverbal stimuli may rely more heavily on perceptual and semantic processing.

## 5 Summary and conclusions

Our literature review (Table 1) highlighted inconsistency in the brain regions associated with the demands on sublexical assembly of articulatory plans. Some studies have proposed that the left dorsal precentral gyrus (dPCg) is involved in sublexical assembly, whereas others have claimed that sublexical assembly is supported by more ventral regions.  Using a multi-factorial design that included object naming conditions as well as word and pseudoword reading and repetition, we associated the demands on sublexical assembly with activation in the anterior part of the left ventral precentral gyrus (vPCg), bordering the left ventral precentral sulcus (vPCs). In contrast, we show that the response in a more dorsal part of the precentral gyrus (dPCg) is more consistent with retrieval effort and demands on executive functioning.

We have also described the contrasting response properties of other left frontal lobe regions that contribute to speech production and compared our interpretation with that of previous studies (Table 6). Of particular interest is the dissociation of two parts of the ventral precentral gyrus: the anterior part associated with sublexical assembly and a more central part that was activated by verbal (words and pseudowords) compared to nonverbal (objects, patterns and humming) stimuli. This motivates future studies using techniques that provide higher spatial resolution (e.g. single-subject data from 7T fMRI) to further investigate the contribution of different vPCg regions to speech production.

Overall, our findings resolve a previous discrepancy in the literature, dissociate three functionally distinct parts of the left precentral gyrus, and refine our understanding of the functional anatomy of speech production.

## Declaration of Competing Interest

The authors declare no competing financial interests.

## Data availability

The data that support the findings of this study are available upon request from the senior author (C.J.P.).

## CRediT authorship contribution statement

**Justyna O. Ekert:** Conceptualization, Writing – original draft, Writing – review & editing, Formal analysis, Visualization. **Diego L. Lorca-Puls:** Investigation, Writing – review & editing. **Andrea Gajardo-Vidal:** Investigation, Writing – review & editing. **Jennifer T. Crinion:** Writing – review & editing. **Thomas M.H. Hope:** Methodology, Writing – review & editing. **David W. Green:** Conceptualization, Writing – review & editing. **Cathy J. Price:** Conceptualization, Methodology, Formal analysis, Writing – original draft, Writing – review & editing, Supervision, Funding acquisition.

## CRediT authorship contribution statement

**Justyna O. Ekert:** Conceptualization, Writing – original draft, Writing – review & editing, Formal analysis, Visualization. **Diego L. Lorca-Puls:** Investigation, Writing – review & editing. **Andrea Gajardo-Vidal:** Investigation, Writing – review & editing. **Jennifer T. Crinion:** Writing – review & editing. **Thomas M.H. Hope:** Methodology, Writing – review & editing. **David W. Green:** Conceptualization, Writing – review & editing. **Cathy J. Price:** Conceptualization, Methodology, Formal analysis, Writing – original draft, Writing – review & editing, Supervision, Funding acquisition.
